# Supplementary material for: Low-temperature and circadian signals are integrated by the sigma factor SIG5
Source: Nat Plants. 2023 Mar 30;9(4):661–72. doi: 10.1038/s41477-023-01377-1 (PMC10119024; doi:10.1038/s41477-023-01377-1)
Supplement: Supplementary file 1 — Reporting Summary [file 41477_2023_1377_MOESM1_ESM.pdf]

nature portfolio

Corresponding author(s): Antony Dodd

Last updated by author(s): May 26, 2022

Reporting Summary

Nature Portfolio wishes to improve the reproducibility of the work that we publish. This form provides structure for consistency and transparency in reporting. For further information on Nature Portfolio policies, see our Editorial Policies and the Editorial Policy Checklist.

Statistics

For all statistical analyses, confirm that the following items are present in the figure legend, table legend, main text, or Methods section.

n/a	Confirmed	
☐	☒	The exact sample size (*n*) for each experimental group/condition, given as a discrete number and unit of measurement
☐	☒	A statement on whether measurements were taken from distinct samples or whether the same sample was measured repeatedly
☐	☒	The statistical test(s) used AND whether they are one- or two-sided<br>*Only common tests should be described solely by name; describe more complex techniques in the Methods section.*
☒	☐	A description of all covariates tested
☒	☐	A description of any assumptions or corrections, such as tests of normality and adjustment for multiple comparisons
☐	☒	A full description of the statistical parameters including central tendency (e.g. means) or other basic estimates (e.g. regression coefficient) AND variation (e.g. standard deviation) or associated estimates of uncertainty (e.g. confidence intervals)
☐	☒	For null hypothesis testing, the test statistic (e.g. *F*, *t*, *r*) with confidence intervals, effect sizes, degrees of freedom and *P* value noted<br>*Give P values as exact values whenever suitable.*
☒	☐	For Bayesian analysis, information on the choice of priors and Markov chain Monte Carlo settings
☒	☐	For hierarchical and complex designs, identification of the appropriate level for tests and full reporting of outcomes
☒	☐	Estimates of effect sizes (e.g. Cohen's *d*, Pearson's *r*), indicating how they were calculated

Our web collection on statistics for biologists contains articles on many of the points above.

Software and code

Policy information about availability of computer code

Data collection	RT-qPCR data were acquired with Bio-Rax CFX v3.1<br>Chlorophyll fluorescence imaging data were acquired and analyzed with Walz ImagingWin v2.47<br>Protein abundance data were acquired and analyzed with ProteinSimple Compass v4.1.0<br>Bioluminescence imaging data were acquired and analyzed with Photek Image32
Data analysis	RNAseq analysis used Fastqc 0.11.3, Trimmomatic 0.33, Kallisto 0.44.0, Degust (DOI: 10.5281/zenodo.3258932; degust.erc.monash.edu).<br>Circadian time-series analysis used the meta2d command within the MetaCycle v1.2.0 package, running in R v4.1.1. Data analysis and graphs prepared using R v4.1.1 (using ggplot2 v3.4.0, ggpubr v0.4.0 and cowplot v1.1.1); RNA sequencing data analyzed using R v3.6.1.

For manuscripts utilizing custom algorithms or software that are central to the research but not yet described in published literature, software must be made available to editors and reviewers. We strongly encourage code deposition in a community repository (e.g. GitHub). See the Nature Portfolio guidelines for submitting code & software for further information.

Data

Policy information about availability of data

All manuscripts must include a data availability statement. This statement should provide the following information, where applicable:

- Accession codes, unique identifiers, or web links for publicly available datasets
- A description of any restrictions on data availability
- For clinical datasets or third party data, please ensure that the statement adheres to our policy

The RNASeq data for this study are available in the European Nucleotide Archive (ENA; https://www.ebi.ac.uk/ena) with the project ID PRJEB45855.

Field-specific reporting

Please select the one below that is the best fit for your research. If you are not sure, read the appropriate sections before making your selection.

☒ Life sciences ☐ Behavioural & social sciences ☐ Ecological, evolutionary & environmental sciences

For a reference copy of the document with all sections, see nature.com/documents/nr-reporting-summary-flat.pdf

Life sciences study design

All studies must disclose on these points even when the disclosure is negative.

Sample size	No sample-size calculation was performed. Sample sizes were based on sample sizes used previously to investigate this signalling pathway (Noordally et al. Science 2013 (https://doi.org/10.1126/science.1230397), Belbin et al. New Phytologist 2016 (https://doi.org/10.1111/nph.14176).
Data exclusions	No data were excluded from analyses.
Replication	At least three completely independent experiments were conducted for each dataset, with the exception of protein abundance experiments where two completely independent experiments were conducted. All replication attempts across all experiments were successful (no data are included that did not replicate successfully).
Randomization	Plant material was positionally-randomized within growth chambers.
Blinding	Blinding was not used for temperature treatments (it is not possible to blind for time of day or temperature treatment, because this is obvious to the experimentalist). Experimentalists were blind to genotype during RNA isolation, RT-qPCR, protein isolation and immunodetection, and during physiological assays (chlorophyll fluorescence analysis, freezing tolerance analysis).

Reporting for specific materials, systems and methods

We require information from authors about some types of materials, experimental systems and methods used in many studies. Here, indicate whether each material, system or method listed is relevant to your study. If you are not sure if a list item applies to your research, read the appropriate section before selecting a response.

Materials & experimental systems

n/a	Involved in the study	
☐	☒	Antibodies
☒	☐	Eukaryotic cell lines
☒	☐	Palaeontology and archaeology
☒	☐	Animals and other organisms
☒	☐	Human research participants
☒	☐	Clinical data
☒	☐	Dual use research of concern

Methods

n/a	Involved in the study	
☒	☐	ChIP-seq
☒	☐	Flow cytometry
☒	☐	MRI-based neuroimaging

Antibodies

Antibodies used	Antibodies used were for PSII D2 (Agrisera AS06146); PSI-C (Agrisera AS10939); RbcL (Agrisera AS03037).
Validation	All three antibodies are validated by Agrisera with this information provided on the supplier's website. Dilution series of antibodies were conducted before experimentation to ensure optimal specificity. PSII D2 (Agrisera AS06146), PSI-C (PSAC) (AS10939) and RbcL (AS03037) validated by manufacturer for use with Arabidopsis thaliana.
